# FuturePall: Forecasting the specialized inpatient palliative care in Germany based on demographic trends and utilization rates

**DOI:** 10.1186/s12904-026-02130-z

**Published:** 2026-06-15

**Authors:** Marcel A. Kamp, Joachim Bergmann, Christiane von Saß, Larissa Fink, Jil Adelstein, Felix Muehlensiepen, Birgitt van Oorschot

**Affiliations:** 1 Brandenburg Medical School Theodor Fontane, Faculty of Health Sciences Brandenburg and Immanuel Clinic, University Hospital of the Brandenburg Medical School Theodor Fontane, Rüdersdorf bei Berlin, Germany; 2 trinovis GmbH, Hannover, Germany; 3 Center for Health Services Research Brandenburg, Brandenburg Medical School Theodor Fontane, Rüdersdorf, Germany; 4 Rangdorf bei Berlin, Germany

**Keywords:** Specialized inpatient palliative care, Future, Prediction, Demographic change

## Abstract

**Introduction:**

Demographic aging poses a significant challenge for specialized inpatient palliative care (SIPC). In the German healthcare system, SIPC is defined as hospital-based multi-professional care delivered through dedicated palliative units, consultation services, or complex care on general wards. To inform future resource allocation, this study predicts the demand for SIPC in Germany up to 2035, specifically examining the impact of shifting population structures on hospital-based palliative service utilization.

**Methods:**

We analyzed baseline utilization data from the 2021 Trinovis Database, which aggregates mandatory structural and performance reports from German hospitals submitted to the Federal Joint Committee (G-BA). Specialized inpatient palliative care (SIPC) was defined using specific Operation and Procedure (OPS) codes. By applying these age-, gender-, and region-specific utilization rates to population projections provided by the German Federal Statistical Office, we forecasted the annual demand for SIPC from the 2021 baseline through the target year of 2035.

**Results:**

Germany’s adult population is expected to decline slightly by 2035, but the proportion of individuals aged 65 years and older will increase. SIPC cases are projected to rise from 108,645 in 2021 to 119,923 by 2035, reflecting a growth rate of 0.7% annually. The proportion of SIPC cases involving patients aged 65 years and older will increase. Regional disparities will persist, with varying growth rates in different federal states.

**Conclusion:**

The “FuturePall” study forecasts a rising demand for SIPC in Germany due to demographic shifts, particularly among older adults. Policymakers must plan for these changes by allocating resources, enhancing infrastructure, and adapting healthcare strategies to meet the evolving needs of an aging population. Future research should explore the impact of non-demographic factors on SIPC demand.

**Supplementary Information:**

The online version contains supplementary material available at 10.1186/s12904-026-02130-z.

## Introduction

The treatment of seriously ill patients with life-threatening conditions remains a significant challenge, burdening both patients and their families with various distressing symptoms. Beyond physical ailments, these patients and their families often experience psychological issues such as anxiety, depression, and existential concerns, as well as social problems like disruptions to family life, social isolation, financial worries, and difficulties with caregiving [1, 2]. Spiritual distress and a loss of inner peace are also common. Palliative medicine is crucial in the care of these patients, aiming to control all forms of burden promptly and improve quality of life. Guidelines advocate for the early integration of palliative care, particularly for patients with advanced oncological diseases, within eight weeks of diagnosis [3–9].

Specialized palliative care in Germany is categorized into distinct inpatient and outpatient sectors focused on acute symptom stabilization [10–12]. Specialized inpatient palliative care is hospital based and delivered through dedicated palliative care units, multi professional consultation services, or complex care on general wards. This form of care plays a vital role within the healthcare framework by providing adequate symptom control and enhancing the quality of life for seriously ill inpatients experiencing a high symptom burden [13, 14]. These services are provided by multi professional teams including palliative care consultants, specialized nurses, physiotherapists, psychotherapists, and clergy [12, 15] In contrast, inpatient hospices are residential non hospital facilities that provide long term end of life care for patients with a limited life expectancy of weeks or months. While specialized inpatient palliative care is indicated for complex medical crises and frequently aims for stabilization or discharge, hospice care is primarily indicated when curative and complex oncological treatments have been concluded in favor of a dignified dying process in a non-acute setting.

In Germany, specialized palliative care is categorized into distinct inpatient and outpatient sectors focused on acute symptom stabilization. Specialized inpatient palliative care is hospital based and delivered through dedicated palliative care units, multi professional consultation services, or complex care on general wards. Specialized inpatient palliative care (SIPC, *spezialisierte stationäre palliativmedizinische Versorgung*) plays a vital role in this framework. Its objective is to provide adequate symptom control and enhance the quality of life for seriously ill inpatients experiencing a high symptom burden [13, 14]. This care is delivered by multi-professional teams, including palliative care consultants and nurses, physio-, psychotherapists, clergy, and other professionals [10, 12, 14–16]. These services can be offered as add-on service in regular hospital wards, dedicated palliative care units, and through palliative medicine consultation services. In contrast, inpatient hospices are residential non hospital facilities that provide long term end of life care for patients with a limited life expectancy of weeks or months. While specialized palliative care is indicated for complex medical crises and often aims for stabilization or discharge, hospice care is primarily indicated when curative and complex oncological treatments have been concluded in favor of a dignified dying process in a non acute setting.

Specialized inpatient palliative care (SIPC, *spezialisierte stationäre palliativmedizinische Versorgung*) plays a vital role in this framework. Its objective is to provide adequate symptom control and enhance the quality of life for seriously ill inpatients experiencing a high symptom burden [13, 14]. This care is delivered by multi-professional teams, including palliative care consultants and nurses, physio-, psychotherapists, clergy, and other professionals [10, 12, 14–16]. These services can be offered as add-on service in regular hospital wards, dedicated palliative care units, and through palliative medicine consultation services.

Like many other countries, Germany is undergoing demographic changes. Life expectancy is increasing, projected to reach 88.1 years for females and 84.4 years for males born in 2060 [17–19]. Concurrently, birth rates remain low. The young adult population has increased due to migration, but the overall average age continues to rise, currently just under 45 years. The “baby boomer” generation, those born post-World War II up to 1964, is now retiring or approaching retirement. This, combined with lower birth rates in subsequent generations and an aging population, is leading to an older and sicker society. Additionally, the gender composition of the population is shifting towards more males, as the generations affected by World War II are dwindling, and in times of prosperity, relatively more boys are born [17–19].

The FuturePall study aims to quantify the projected utilization-based demand for specialized inpatient palliative care in Germany through 2035 by analysing age, gender, and region-specific demographic trends to identify future capacity requirements and geographic disparities within the changing demographic landscape.

## Methods

### Ethics approval, data availability, data security and study design

This study utilized exclusively anonymized and aggregated data derived from statutory hospital reports in accordance with the ethical principles of the Helsinki Declaration. The analysis integrated public records submitted under § 136b SGB V. At no stage was person-level or identifiable patient information accessible or utilized. The institutional and local ethics committee of the Brandenburg Medical School (Study ID: 190032024-ANF, Germany) approved the study protocol. Furthermore, the reporting of this study complies with the STROBE guidelines for observational studies [20].

### Study design, setting, and data source

Our study and projections are based on the trinovis VISION database (trinovis GmbH, Hanover, Germany). This database is a comprehensive repository that integrates structural and performance data from the mandatory annual Quality Reports submitted by all German hospitals to the Federal Joint Committee (G-BA) under § 136b SGB V. Consequently, the data represents a near-complete census of the German hospital landscape. As submission is a legal requirement for all hospitals the database provides a near-complete census (approx. 99%) of the national hospital landscape. Furthermore, the database incorporates case-level data according to § 21 KHEntgG, public population statistics (demographics, diagnoses, and procedures), and specific geographic data. This multi-layered approach allows for the synchronization of clinical performance data with regional population projections. Data extraction was performed via structured queries within the trinovis VISION platform. This process utilized the platform’s integrated tools to aggregate the § 136b SGB V quality reports and § 21 KHEntgG case-level statistics into the stratified utilization rates used for modeling. For the 2021 baseline year, the trinovis VISION database included 1,849 hospitals, 468,548 beds, and 15,832,663 cases.

### Cohort and study size

The baseline dataset comprises all adult inpatient cases (≥ 18 years) documented within the mandatory 2021 statutory reports submitted by German hospitals to the Federal Joint Committee (G-BA). This reference year provides the empirical foundation for established palliative care utilization rates, which were subsequently modelled against population projections through 2035 to forecast future demand. Inclusion in the cohort required the presence of at least one specialized palliative care procedure code (*Operation and Procedure Code*; OPS: 8-982, 8-98e, or 8-98 h). Patients under the age of 18 were excluded to ensure the analysis focused strictly on the adult SIPC framework, which operates under distinct structural and billing regulations.”

### Definitions and variables

The primary objective of this study was to project the future demand for SIPC in Germany based on demographic shifts. This analysis exclusively considers facilities operating within the German Diagnosis Related Groups-System (DRG), which serves as the official classification for encoding medical interventions.

SIPC was defined using three specific OPS codes:

- 8-982 (Complex palliative care): Specialist management and comprehensive care, e.g. provided on regular hospital wards.
- 8-98e (Specialized palliative care on a palliative care ward): Treatment within an independent, dedicated palliative care unit with 24/7 medical on-call service.
- 8–98 h (Specialized palliative care consultation): Support provided by a multi-professional palliative care consultation service for patients on other specialist wards.

The demand for care was estimated using the “OPS case number expectation value.” To ensure high granularity, we first established baseline utilization rates (γ) for the year 2021. These rates were stratified by OPS code (i), age group (a), gender (g), and regional unit (r):

$$\upgamma_{\text{i, a, g, r}} = \text{OPS cases}_{\text{i, a, g, r}}/ \mathrm{population}_{\text{a, g, r}}$$

To forecast future demand for a specific target year (t), the OPS case number expectation value (Projected Case Demand) was derived by multiplying these stratified baseline rates by the corresponding population projections (P) provided by the German Federal Statistical Office (Statistisches Bundesamt):

$$\text{Projected Case Demand}_{\mathrm{t}} \sum (\upgamma_{\text{i, a, g, r}} \times \mathrm{P}_{\text{a, g, r, t}})$$

This model assumes that age- and gender-specific utilization rates remain constant, isolating the impact of demographic change, specifically the aging “baby boomer” cohorts, on SIPC requirements. For predicting demographic changes of the German population, we used the average of two moderate variants from the 14th Coordinated Population Projection [21]. Both variants (G2-L2-W1 and G2-L2-W2) assume a moderate birth rate of 1.55 children per woman and a moderate increase in life expectancy, projecting a life expectancy at birth in 2060 of 84.4 years for males and 88.1 years for females. The variants differ in their annual net migration assumptions: Variant 1 (W1) assumes a long-term net migration averaging 147,000 people per year, while Variant 2 (W2) assumes an average of 221,000 people per year. Clinical case numbers from the 2021 baseline were applied to these demographic frameworks for all subsequent analyses.

### Statistics

Data were organized using Microsoft Excel for Mac (Version 16.78, Microsoft Corporation, Redmond, Washington, USA). Descriptive statistical analyses and graphing were conducted using GraphPad Prism 9 for macOS (Version 9.5.0, GraphPad Software, Inc., La Jolla, USA).

**Fig. 1:**
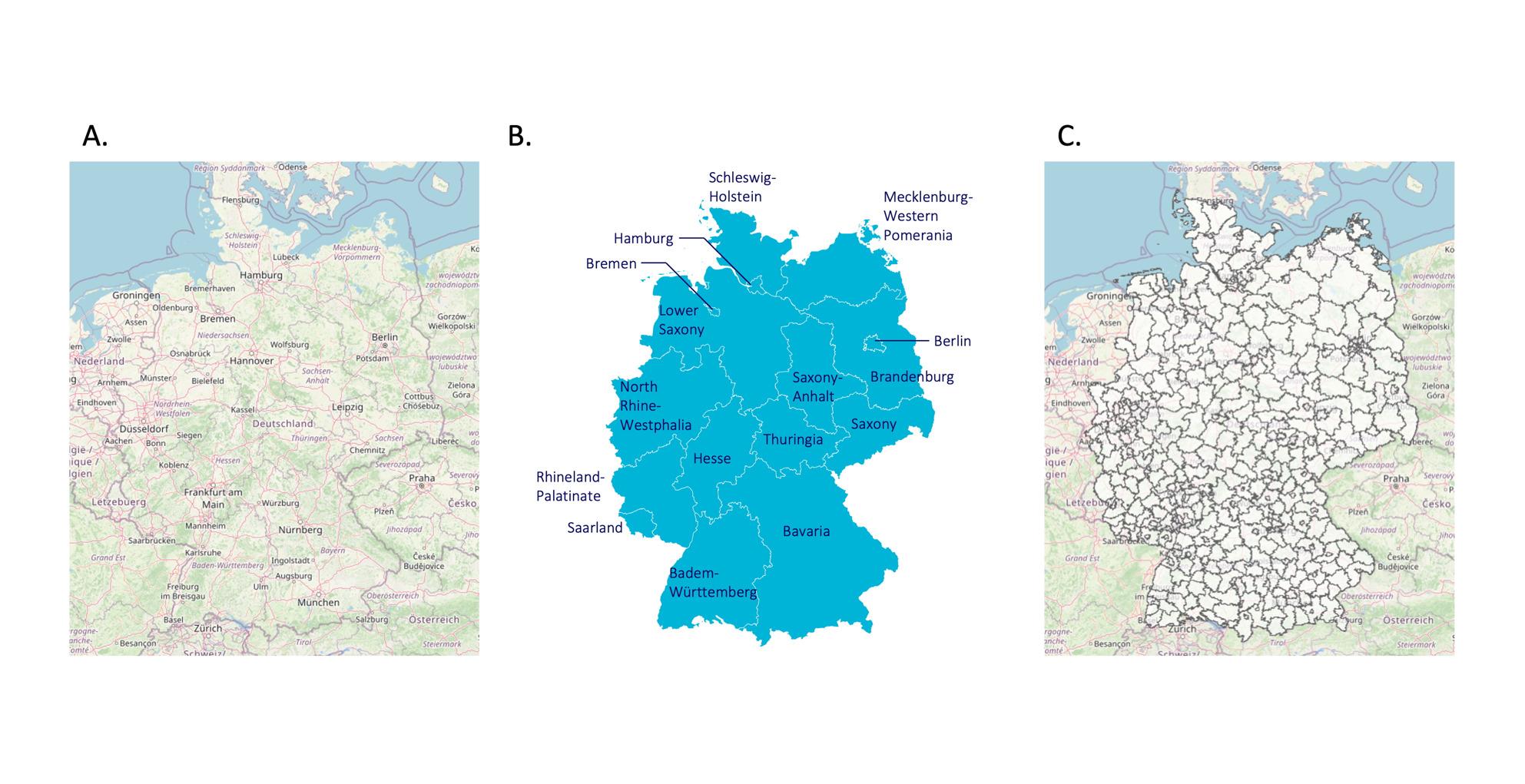
Administrative and regional structure of the study area. **A** shows the geographic loca-tion of Germany and figure **B**. the 16 federal states (Bundesländer), which serve as the primary level of regional healthcare governance. **C** shows the over 400 districts (Landkreise and kreisfreie Städte), representing the high-resolution administrative units used for the localized projection of specialized inpatient palliative care (SIPC) demand

Geographic analysis was conducted across two hierarchical administrative levels in Germany: the 16 federal states (Bundesländer) and the 400 districts (Landkreise und kreisfreien Städte, Fig. 1). These regional units serve as the structural basis for healthcare planning and allow for the identification of spatial disparities in projected palliative care demand.

## Results

### Current and projected demographic evolution of the adult population in Germany

In 2021, Germany had 69,373,865 citizens over the age of 18 years, comprising 83.3% of the total population (suppl Fig. 1). Among them, 18,436,499 were aged 65 years and older (22.1%), and 6,111,655 were aged 80 and older (7.3%).

**Fig. 2:**
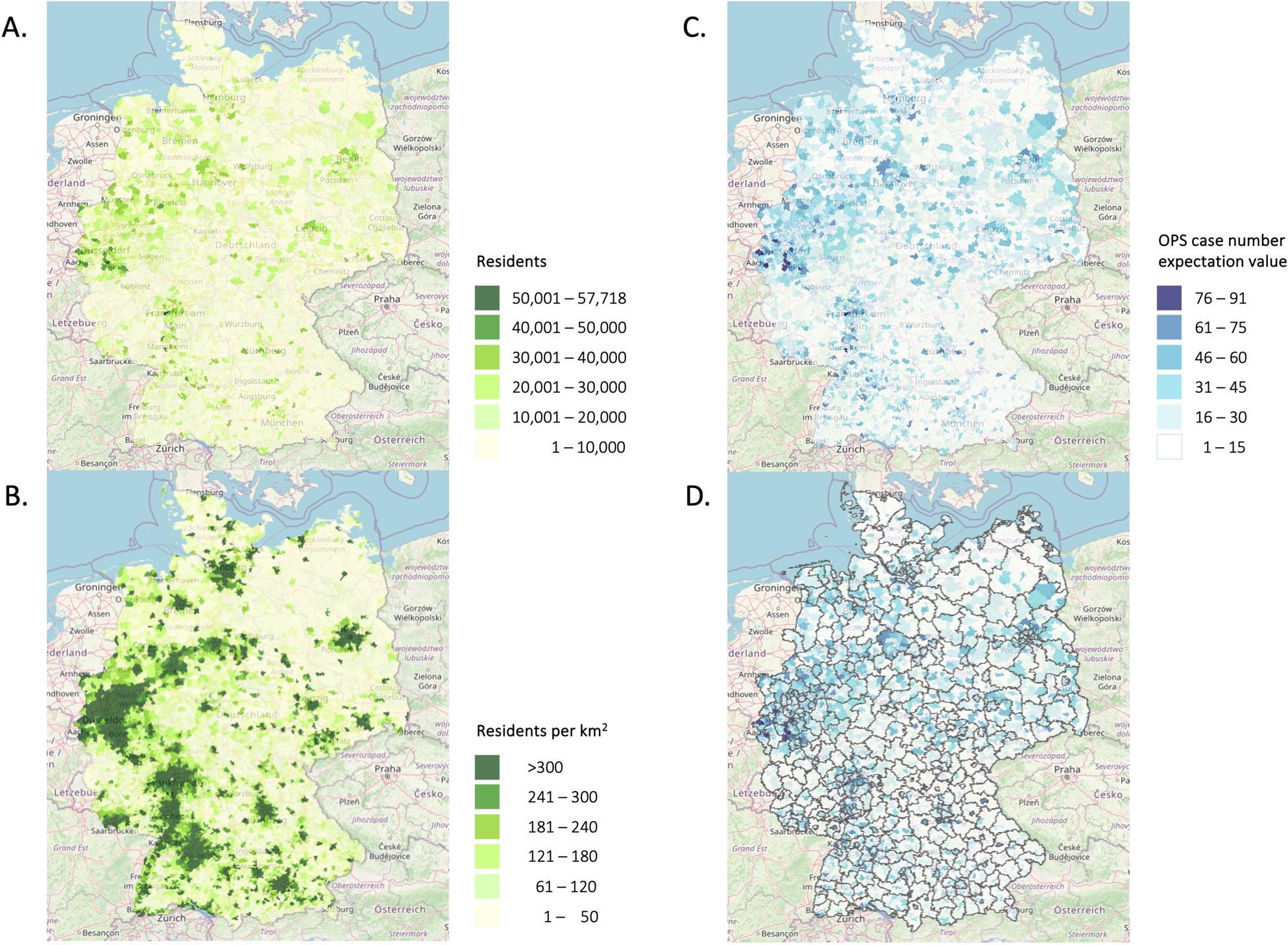
Forecast of population and specialized palliative care needs in Germany. **A** shows a forecast of the German population in 2035 per postcode area. **B** displays the projected density of residents per square kilometer per postcode area. **C** and **D** present the OPS case number expectation value per postcode area, providing an overview of the need for specialized palliative care in 2035 based on demographic factors. Figure 2D specifically shows the boundaries of the districts depicted in Figure 2C

By 2035, the adult population in Germany is projected to be 68,983,988, a decrease of 0.6% (suppl. Figure 1). The proportion of those aged 65 years and older will rise to 27.8% (9,601,842 people), while those aged 80 years and older will account for 8% (3,875,950 people). Regional differences will be pronounced across the federal states (suppl. Figure 1, Fig. 2A.): Berlin is expected to see the highest population growth at 6.2%, while Saxony-Anhalt will experience the largest decline at 12.2%. On the district level, Vechta in Lower Saxony is projected to grow by 26%, whereas Suhl in Thuringia is expected to see a 14% decrease.

### Specialized inpatient palliative care utilization in 2021 and projections through 2035

**Fig. 3:**
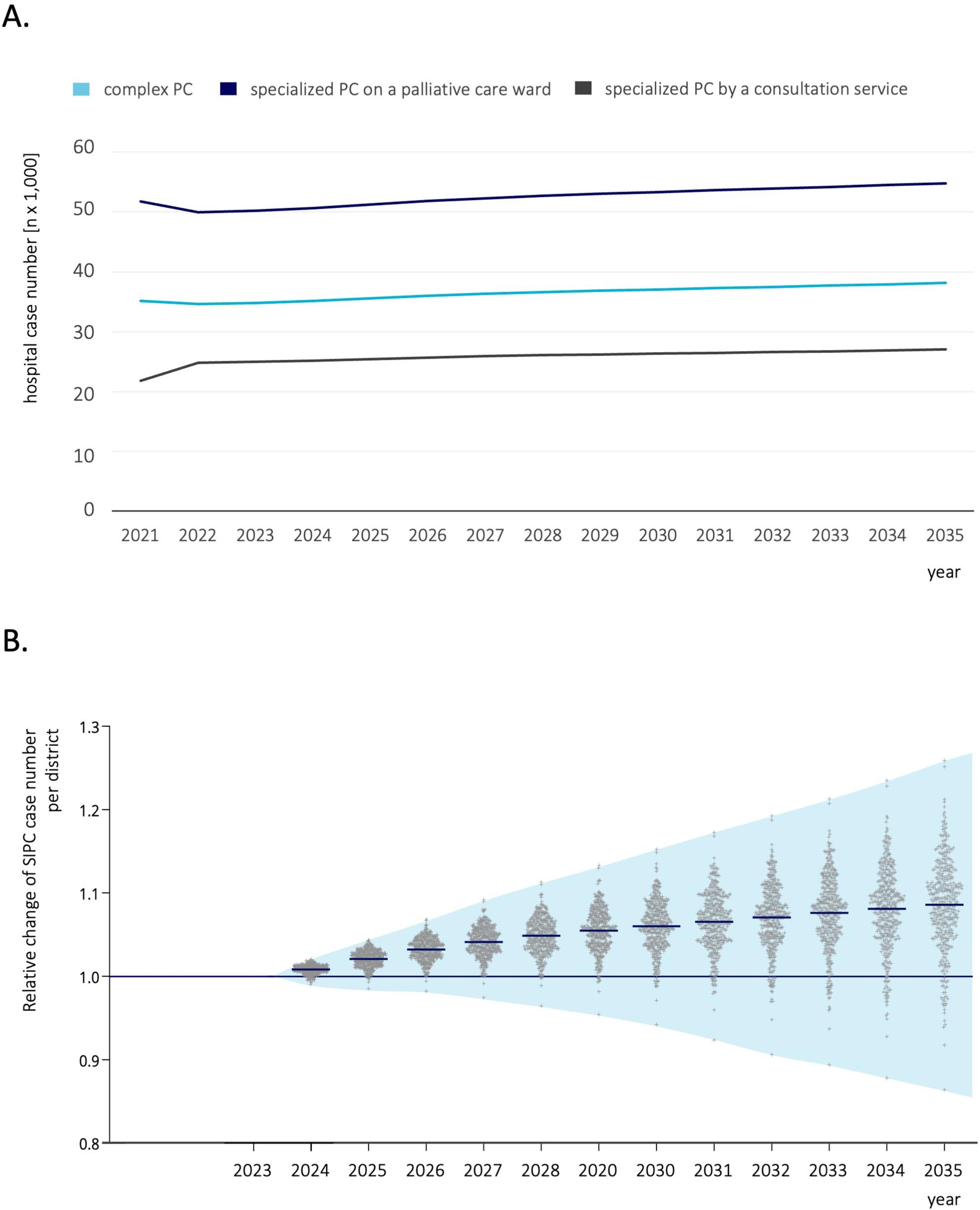
Projected development of specialized inpatient palliative care in Germany. **A** provides an overview of the projected development of SIPC cases across Germany, while **B** illustrates the relative development of SIPC cases in each regional district

In 2021, there were 108,645 SIPC cases, with a rate of 711 cases per 100,000 adult hospital cases (0.71%). This included 35,121 cases (32.3%) of complex palliative care, 51,726 cases (47.6%) on palliative care units, and 21,798 cases (20%) through specialized consultation services (Fig. 3).

By 2035, the expected number of SIPC cases is projected to rise to 119,923, growing at an average annual rate of 0.7% (tabl. 1; Fig. 3). The rate SIPC cases will slightly increase to 744 per 100,000 hospital cases. The expected number of complex palliative care cases will rise to 38,128 (31.8%), palliative care unit cases to 54,775 (45.7%), and specialized consultation cases to 27,020 (22.5%), with growth rates of each 0.7% for complex and specialized palliative care on a palliative care unit, and 0.6% for consultation services.

### Regional Differences in specialized in-patient palliative care

**Fig. 4:**
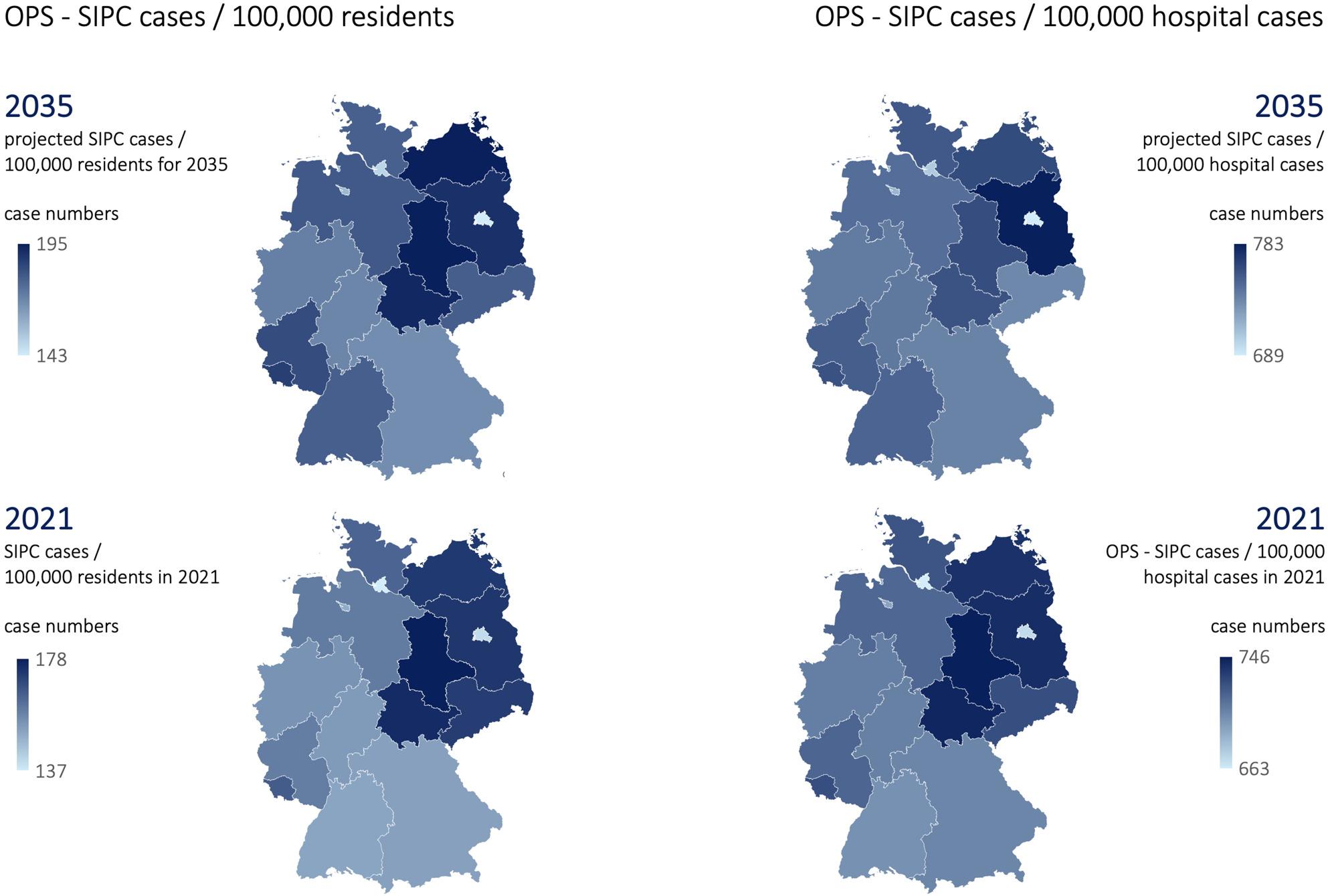
Regional differences in specialized inpatient palliative care. Figure 4 gives an overview over the predicted specialized palliative care cases per 100,000 inhabitants (A./C.) and the predicted specialized palliative care cases per 100,000 hospital cases (B./D.) in 2021 and 2035

In 2021, there were notable regional differences in SIPC. The number of cases per 100,000 adult residents ranged from 137 in Hamburg to 179 in Saxony-Anhalt, and from 663 to 746 per 100,000 adult hospital patients (Fig. 4; tabl. 2; suppl. tabl. 1).

Projections for 2035 indicate persistent regional disparities. The demand will vary by state, with changes ranging from − 0.2% in Saxony-Anhalt to 0.9% in Lower Saxony and Baden-Württemberg (tabl. 2). The rate of SIPC cases will range from 689 per 100,000 hospital cases in Berlin to 783 in Brandenburg (Fig. 4, suppl. tabl. 1). The projected SIPC need per district and postal code regions are shown in Fig. 2.

### Age- and gender-specific SIPC utilization in 2021 and projections through 2035

**Fig. 5:**
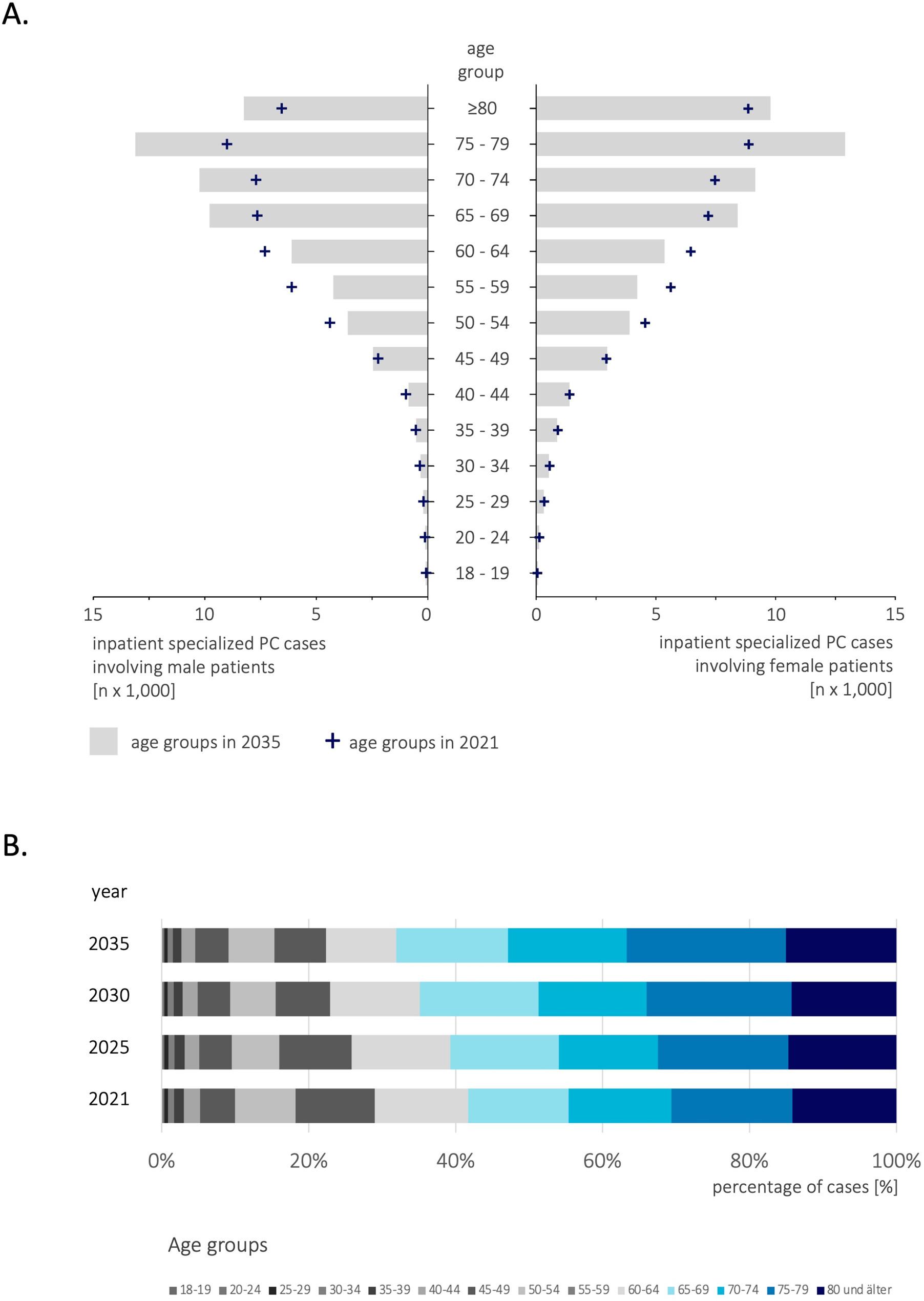
Forecast of the age and sex distribution of specialized palliative care cases in Germany. **A** shows the 2021 and projected 2035 age and sex distribution of specialized palliative care cases in Germany. **B** presents the relative age distribution in 2021 and the projected relative age distributions in 2025, 2030, and 2035

In 2021, 51% (55,405) of the 108,645 palliative care cases were female. 41.7% (45,292) of cases involved patients aged 18–64 years, and 58.3% (63,353) involved patients aged 65 years and older, including 15,418 cases (14.2%) aged 80 years and older (tabl. 3; Fig. 5).

By 2035, the proportion of female cases is projected to be 50.1% (60,060 out of 119,923 cases). In parallel, the proportion of cases involving patients aged 18–64 years is expected to decrease to 31.9% (38,312), while cases involving patients aged 65 years and older will rise to 68.1% (81,611), with 15% (18,033) aged 80 years and older (tabl. 3; Fig. 5).

## Discussion

In alignment with our study aims, our analysis of the changing demographic landscape yields the following core findings:

- **Quantitative Demand Trajectory**: We project a significant 10.4% increase in the absolute volume of SIPC cases by 2035 (from 108,645 cases in 2021 to 119,923 cases in 2035), indicating that current inpatient capacities will face sustained upward pressure.
- **Demographic Shift**: The patient profile will undergo a profound shift toward older age groups, with the 65 + cohort accounting for 68.1% of total cases. Notably, the “oldest-old” (≥ 80 years) will reach 15.0% of the total volume, with a marked proportional increase in male patients within this advanced age group, necessitating age- and gender-sensitive care models.
- **Geographic Disparities**: Based on a status-quo projection, divergent demographic trends (annual growth − 0.2% to 0.9%) will likely perpetuate existing geographic disparities within current supply-driven structures.

Our study estimates the future need for SIPC due to demographic changes. We calculated the OPS case number expectation value by multiplying the quota with the number of inhabitants per ICD/OPS, age group, gender, and zip code. Our findings indicate an increasing demand for SIPC, particularly among patients aged 65 years and older. Current literature lacks models that project palliative care needs in Germany. However, studies in other domains offer some parallels. For instance, projections for prostate cancer incidence and radiotherapy needs suggest a 27.4% increase due to demographic changes (Sonnhoff et al., 2023). Estimates for palliative care needs in Great Britain predict a 35–42% increase by 2040, driven by higher case numbers of dementia and cancer (Etkind et al., 2017). Scotland and Ireland also expect significant increases in palliative care demand by 2040 and 2046, respectively (Finucane et al., 2018, 2019; May et al., 2019). Our study also highlights regional disparities in Germany’s future population development and SIPC usage. Data from 145,372 patients insured with BARMER health insurance, who died between 2016 and 2019, revealed significant regional differences in palliative care provision (Freytag et al., 2023). Inpatient palliative care was provided to 27.2% of deceased patients nationwide, with the lowest rate in Lower Saxony (21.5%) and the highest in Thuringia (37.4%). These disparities also extended to the place of death, timing of palliative care involvement, and quality-related outcome indicators. While this study focuses on demographic projections, the observed regional disparities in SIPC warrant structural contextualization. The observed regional disparities in SIPC utilization can only partially be explained by objective medical need or patient characteristics, while socioeconomic factors in Germany play a subordinate role compared to international findings [22–24]. Instead, utilization likely follows a supply-sensitive care model, largely dependent on local infrastructure density and historically developed care structures. These disparities align with Penchansky and Thomas’s theory of access, which suggests that utilization is determined less by need than by the spatial availability and accessibility of services and the organizational maturity of regional networks [25]. In rural areas, longer travel distances and lower facility density result in shorter care durations and higher barriers, particularly for nursing home residents [26, 27]. Furthermore, differences in the place of death and the timing of palliative involvement reflect the varying implementation of policy interventions, such as the Hospice and Palliative Care Act (HPG) or Advance Care Planning (ACP) [10] (Tables 1, 2 and 3).

**Table 1:** Forecast of specialized inpatient palliative care cases in Germany

Specialized palliative care treatment	2021	2022	2023	2024	2025	2026	2027	2028	2029	2030	2031	2032	2033	2034	2035	Annual growth rate
8-982 complex palliative care treatment	35,121	34,610	34,838	35,117	35,559	35,991	36,328	36,618	36,862	37,081	37,295	37,508	37,722	37,925	38,128	0.7 %
8-98e specialized palliative care treatment on a palliative care ward	51,726	49,910	50,226	50,613	51,228	51,828	52,295	52,692	53,025	53,323	53,617	53,911	54,206	54,491	54,775	0.7 %
8-98h specialized palliative care treatment by a consultation service	21,798	24,862	25,003	25,176	25,452	25,717	25,922	26,092	26,233	26,361	26,489	26,620	26,754	26,885	27,020	0.6 %
Total specialized palliative care	108,645	109,382	110,067	110,906	112,238	113,536	114,545	115,402	116,120	116,765	117,401	118,039	118,682	119,302	119,923	0.7 %

**Table 2:** Forecast of population and specialized inpatient palliative care need in the Federal States of Germany

Year	Germany	Baden-Württemberg	Bavaria	Berlin	Brandenburg	Bremen	Hamburg	Hesse	Mecklenburg-Western Pomerania	Lower Saxony	North Rhine-Westphalia	Rhineland-Palatinate	Saarland	Saxony	Saxony-Anhalt	Schleswig-Holstein	Thuringia
Baseline population																	
2021	15291956	1991498	2368398	640509	497508	121814	317511	1133261	318450	1483498	3254980	759156	190160	801783	439246	553620	420563
Projected population																	
2025	15471216	2009838	2402718	670487	497581	125143	322513	1149571	320169	1511073	3296110	764457	188944	805647	430433	559163	417368
2030	15763941	2055332	2468803	674457	503230	127240	329194	1177471	326813	1562272	3364215	780160	189207	804480	421071	565243	414753
2035	16127474	2115750	2539511	674457	503230	127240	337694	1211634	337022	1629060	3454465	795269	190725	809076	414971	571829	415541
Baseline SIPC case numbers																	
2021	108645	13942	16685	4290	3685	843	2104	8008	2353	10632	23025	5448	1382	5827	3278	4011	3130
Projected population																	
2025	112238	14562	17349	4530	3761	883	2172	8342	2426	11076	23645	5677	1417	5875	3252	4117	3155
2030	116765	15268	18076	4650	3942	909	2269	8667	2560	11627	24747	5893	1443	6015	3264	4225	3209
2035	119922	15909	18740	4650	3942	909	2380	8996	2571	12173	25604	6002	1450	5952	3162	4327	3156

**Table 3:** Forecast of the age distribution of specialized inpatient palliative care cases in Germany

2021					
Age group	Residents	OPS case number expectation value			
		8-982	8-98e	8-98h	Total
Total (18 - 64 years)	50,937,366	14,413	21,348	9,531	45,292
18-19 years	1,570,656	32	72	25	129
20-24 years	4,522,527	70	92	101	263
25-29 years	4,892,729	124	232	175	531
30-34 years	5,553,604	239	432	252	923
35-39 years	5,335,194	448	650	346	1,444
40-44 years	5,132,299	704	1,093	584	2,381
45-49 years	4,864,404	1,697	2,357	1,113	5,167
50-54 years	6,225,112	2,768	4,276	1,903	8,947
55-59 years	6,846,797	3,800	5,557	2,376	11,733
60-64 years	5,994,044	4,531	6,587	2,656	13,774
Total (≥65 years)	18,436,499	20,708	30,378	12,267	63,353
65-69 years	4,967,930	4,968	7,063	2,817	14,848
70-74 years	4,173,292	4,950	7,270	2,966	15,186
75-79 years	3,183,622	6,017	8,556	3,328	17,901
≥80 years	6,111,655	4,773	7,489	3,156	15,418
Total	69,373,865	35,121	51,726	21,798	108,645
2035					
Age group	Residents	OPS case number expectation value			
		8-982	8-98e	8-98h	total
Total (18 - 64 years)	45,916,218	11,612	17,459	9,241	38,312
18-19 years	1,675,765	27	84	68	179
20-24 years	4,104,343	59	105	81	245
25-29 years	4,302,039	134	219	173	526
30-34 years	4,587,972	174	405	283	862
35-39 years	5,105,796	349	625	429	1,403
40-44 years	5,379,175	650	1,058	552	2,261
45-49 years	5,572,154	1,608	2,484	1,333	5,425
50-54 years	5,269,837	2,334	3,402	1,753	7,489
55-59 years	4,936,704	2,625	3,839	1,992	8,456
60-64 years	4,982,433	3,652	5,238	2,576	11,466
Total (≥65 years)	23,067,770	26,516	37,317	17,778	81,611
65-69 years	6,036,793	5,926	8,307	3,959	18,193
70-74 years	5,779,184	6,369	8,968	4,045	19,382
75-79 years	4,560,027	8,449	11,918	5,637	26,004
≥80 years	6,691,766	5,771	8,124	4,137	18,033
Total	68,983,988	38,128	54,775	27,020	119,923

While this study focuses on specialized inpatient palliative care (SIPC), it represents only one component of the specialized palliative framework alongside specialized outpatient palliative care (SAPV). Utilization in Germany is characterized by supply-sensitive demand. Case numbers are driven more by regional infrastructure and capacity than by clinical need or patient-related factors [22, 23, 28]. Although the vast majority of the population can access both service types within 30 minutes, significant regional disparities persist [24, 29, 30]. These structural variances lead to notable substitution effects: a high density of SAPV units correlates with increased outpatient case numbers, whereas higher inpatient capacity drives SIPC utilization [22, 23, 28]. Consequently, the mutual influence of these sectors reflects local service availability rather than systematic clinical integration. While it is often hypothesized that expanding outpatient services could mitigate inpatient demand, the supply-sensitive nature of the German system suggests that SAPV and SIPC may instead function as complementary sectors that address distinct levels of clinical complexity. Consequently, an expansion of outpatient infrastructure may not necessarily offset the projected 10.4% increase in SIPC cases, as both sectors likely uncover different areas of previously unmet need within the aging population. Nevertheless, regions with integrated networks achieve superior outcomes, including higher rates of home deaths and fewer invasive end-of-life measures [31–33]. Additionally, integrated oncology-palliative care models might additionally enhance patient quality of life, symptom control, and psychosocial wellbeing, particularly when palliative care is introduced early and systematically alongside standard oncology care (e.g., the integrated EMSO-designated centres, “Comprehensive Cancer Centre” models in Germany or the ‘Gold Standards Framework’ in the UK) [14, 15, 34–36]. For our “FuturePall” model, these findings suggest that while demographic shifts remain the primary driver of demand, the actual realization of SIPC cases will be contingent upon the local evolution of palliative infrastructure.

The regional disparities identified in our model must be viewed alongside the ongoing German hospital reform. While centralization of hospital care may result in only a marginal reduction of palliative care facilities, case numbers and travel times, the projected 10.4% increase in cases suggests that the primary future challenge will be ensuring sufficient bed capacity and staffing within existing structures to meet the rising demand of the oldest-old population [24]. This aligns with international patterns in the UK and Canada, where regional infrastructure, rather than patient need, remains the primary driver of service utilization [37–39]. Our findings thus highlight a distinctive global challenge: the persistence of supply-driven disparities that complicate the management of rising demand in aging societies.

Beyond age, the projected masculinization of the elderly population may significantly influence SIPC utilization. While women on average live longer and are thus more likely to require end of life care, the observed gender differences are particularly evident in the higher utilization by women aged 75 and older. This trend may be driven by differences in informal support systems [40, 41]. In Germany, older men are more frequently cared for by their partners at home. Women often outlive their spouses due to higher life expectancy and consequently live alone [40, 41]. This leads to a higher necessity for inpatient stabilization when outpatient support is unavailable. Furthermore, evidence suggests that gender is a key determinant in healthcare uptake. Men utilize specialized palliative care less frequently and show lower preferences for such interventions [42–44]. While general hospitalization rates are gender neutral, women more frequently utilize non acute and supportive services [45]. Consequently, a demographic shift toward more males might lead to a relative decrease in SIPC utilization or an increase in unmet needs. Future care planning must therefore address these gender specific social barriers to ensure equitable access as the population age structure shifts.

The focus on specialized inpatient palliative care is particularly relevant given the current structural shifts in German healthcare. Although health policy and patients strongly favour outpatient stabilization, SIPC remains indispensable for managing high-intensity medical crises, refractory symptoms, and complex psychosocial distress that exceed the capacity of home-based services or general palliative wards. Furthermore, as the oldest old population grows, the lack of informal caregiving structures in single person households likely maintains or even increases the necessity for hospital based palliative resources.

To further refine our understanding of how demographic developments will impact not only palliative care but also broader oncology case numbers, an in-depth analysis of billing data and future projections is essential. By continuously monitoring OPS figures, healthcare systems can be better equipped to design inpatient palliative care services that are responsive to evolving needs. Additionally, engaging in qualitative studies with healthcare professionals and policymakers may be used for formulating actionable recommendations that will shape the future of SIPC. Importantly, there remains a significant research gap in evaluating the quality of both inpatient and outpatient palliative care. Exploratory studies addressing this gap are urgently needed to ensure that care standards improve in parallel with rising demand. Ultimately, these initiatives will help drive the development of a palliative care infrastructure that not only meets the growing demand but also ensures equitable, high-quality care for all patients.

### Limitations

1. In Germany, not all palliative care units use the DRG system for billing. About 70 palliative care units are classified as “special facilities” (besondere Einrichtungen), each requiring at least 5 beds, with an average of 8 beds per unit. An 8-bed unit typically manages around 250 inpatient cases annually, amounting to approximately 17,500 cases across all such facilities. These units negotiate daily rates for palliative care services directly with health insurers, so their services are not included in hospital statistics. Therefore, the need for SIPC is likely underreported, and the actual demand is probably higher.
2. Moreover, we are unable to determine the need for specialized outpatient palliative care as these data are not routinely collected. Outpatient palliative care, especially in the context of chronic, progressive illnesses, plays a crucial role in patient management, symptom control, and quality of life enhancement. Its omission from this study potentially overlooks a significant component of the care continuum for these patients.
3. The projection model is based on a status-quo assumption. We assume that the stratified utilization rates (γ) calculated for the baseline year 2021 remain constant until 2035. This approach isolates the impact of demographic change, specifically the transition of the ‘baby boomer’ cohorts into high-utilization age groups, from other potential variables such as changes in clinical guidelines, coding behaviour (OPS), or healthcare legislation.
4. Our projection relies on structural demographic shifts. However, future SIPC needs will also be influenced by non-demographic factors. The rate of specialized palliative care usage could change over time. Currently, 34.8% of deceased BARMER policyholders received some form of palliative care [23]. However, not all patient groups with life-threatening illnesses and symptom burden have easy access to specialized palliative care. For instance, only in 10.5% of German hospital cases between 2019 and 2022 involving malignant glioma patients, palliative care was utilized, rising to 40.6% among those who died in the hospital [43]. In high-mortality areas like intensive care, palliative care co-treatment rates are likely even lower [46]. Our model does not account for potential changes in palliative care utilization rates driven by new studies and guidelines.
5. Another critical factor is how increased life expectancy affects illness and morbidity. Extended life expectancy may either prolong the period of morbidity, representing a “failure of success,” or delay the onset of morbidity, as suggested by the “compression of morbidity” model [47, 48]. Our model does not consider changes in the morbidity period resulting from increased life expectancy, which may lead to inaccuracies in our projections.
6. Additionally, inpatient care planning in Germany is set to undergo significant reforms, aiming to shift previously inpatient services to outpatient settings, specialize hospitals, reduce hospital numbers, and plan inpatient services based on quality criteria [49]. These structural reforms will undoubtedly impact future case numbers, but the extent of these changes remains difficult to quantify. Our analysis does not include potential alterations due to political and regulatory changes.

## Conclusion

The FuturePall study demonstrates that demographic aging alone will drive a 10.4% increase in SIPC demand by 2035, with a profound shift toward the ‘oldest-old’ and male patient cohorts. These findings reveal that maintaining the status quo will be insufficient. Instead, policymakers must address the persistent supply-driven regional disparities to ensure equitable access. Ultimately, our findings suggest that future healthcare strategies must move beyond current supply patterns to proactively align SIPC infrastructure with the shifting demographic landscape of an aging society.

## Supplementary Information

Supplementary Material 1.

Supplementary Material 2.

## Data Availability

The dataset supporting the conclusions of this article is included within the article and its additional file (supplements). Data was acquired using the publicly available data of the Federal Joint Committee (Gemeinsamer Bundesausschuss, G-BA).
