# Supplementary figures and images for: FuturePall: Forecasting the specialized inpatient palliative care in Germany based on demographic trends and utilization rates
Source: BMC Palliat Care. 2026 Jun 15;25:172. doi: 10.1186/s12904-026-02130-z (PMC13267715; doi:10.1186/s12904-026-02130-z)

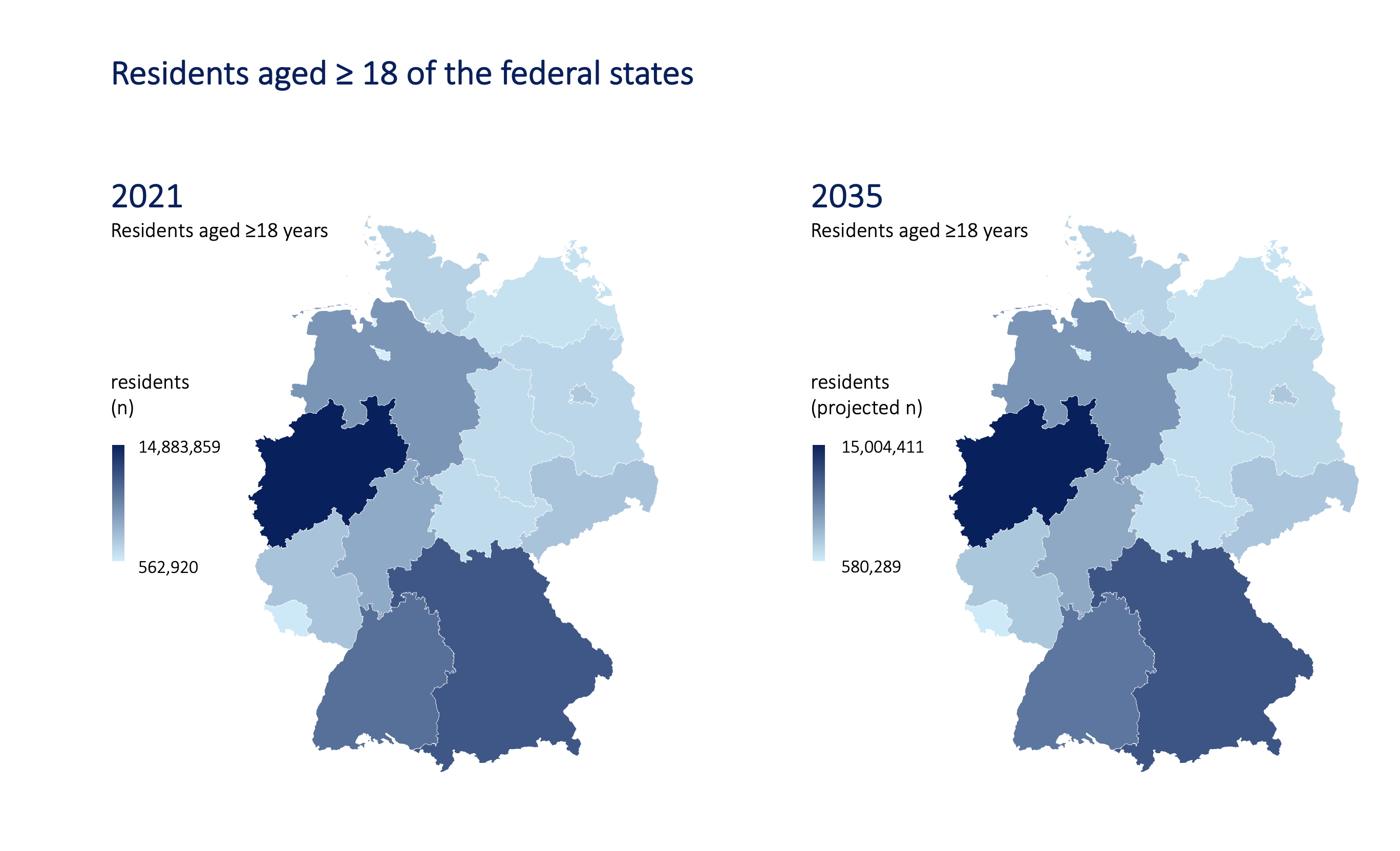

Supplement: Supplementary file 2 — Supplementary Material 2. [file 12904_2026_2130_MOESM2_ESM.jpg]
